# Basic and complex emotion recognition in children with autism: cross-cultural findings

**DOI:** 10.1186/s13229-016-0113-9

**Published:** 2016-12-19

**Authors:** Shimrit Fridenson-Hayo, Steve Berggren, Amandine Lassalle, Shahar Tal, Delia Pigat, Sven Bölte, Simon Baron-Cohen, Ofer Golan

**Affiliations:** 1 Department of Psychology, Bar-Ilan University, Ramat-Gan, Israel; 2 Center of Neurodevelopmental Disorders (KIND), Department of Women’s and Children’s Health, Karolinska Institutet, Stockholm, Sweden; 3 Autism Research Centre, Department of Psychiatry, University of Cambridge, Cambridge, UK; 4 Athinoula A. Martinos Center for Biomedical Imaging, Massachusetts General Hospital, Harvard Medical School, Charlestown, MA USA; 5 Center of Psychiatry Research, Stockholm County Council, Stockholm, Sweden

**Keywords:** Autism spectrum condition, Emotion recognition, Basic emotions, Complex emotions, Cross-cultural research

## Abstract

**Background:**

Children with autism spectrum conditions (ASC) have emotion recognition deficits when tested in different expression modalities (face, voice, body). However, these findings usually focus on basic emotions, using one or two expression modalities. In addition, cultural similarities and differences in emotion recognition patterns in children with ASC have not been explored before. The current study examined the similarities and differences in the recognition of basic and complex emotions by children with ASC and typically developing (TD) controls across three cultures: Israel, Britain, and Sweden.

**Methods:**

Fifty-five children with high-functioning ASC, aged 5–9, were compared to 58 TD children. On each site, groups were matched on age, sex, and IQ. Children were tested using four tasks, examining recognition of basic and complex emotions from voice recordings, videos of facial and bodily expressions, and emotional video scenarios including all modalities in context.

**Results:**

Compared to their TD peers, children with ASC showed emotion recognition deficits in both basic and complex emotions on all three modalities and their integration in context. Complex emotions were harder to recognize, compared to basic emotions for the entire sample. Cross-cultural agreement was found for all major findings, with minor deviations on the face and body tasks.

**Conclusions:**

Our findings highlight the multimodal nature of ER deficits in ASC, which exist for basic as well as complex emotions and are relatively stable cross-culturally. Cross-cultural research has the potential to reveal both autism-specific universal deficits and the role that specific cultures play in the way empathy operates in different countries.

## Background

Autism spectrum conditions (ASC) are neurodevelopmental conditions characterized by social communication and interaction difficulties, circumscribed interests, and a preference for sameness and repetition. Individuals with ASC experience difficulties processing and interpreting socio-emotional cues [1–3]. These emotion recognition (ER) difficulties are recognized as part of the social communication challenge, characteristic of ASC [4] and predict adaptive socialization skills [5]. The recognition of others’ emotions and mental states relies on the integration of emotional cues from various channels. These include facial expressions, vocal intonation, and body language, as well as contextual information [6].

Due to its centrality in emotional expression, the majority of ER research has focused on facial expression. In typical development (TD), the skills necessary to recognize and discriminate between emotional expressions are present from infancy [7, 8]. Throughout development, facial ER improves, with children gradually becoming “emotion experts” [9]. In contrast, children and adults with ASC show reduced attention to faces and facial expressions [10–13]. Altered attention, specifically to the eye region of the face [14, 15], has a significant effect on facial affect recognition in individuals with ASC [16]. In addition, children with ASC process faces in a piecemeal fashion rather than holistically [17–19] (see [20], for a different perspective). Consequently, children with ASC show reduced “face expertise” [11].

Some studies of facial ER have reported deficits in participants with ASC [21–24]. Other studies, however, have found facial emotion recognition skills in children with ASC to be equal to those in TD peers [25–28]. Recent review of facial expression in ASC had proposed that these differences in documented ER are dependent on participant demographics, task selection, and stimulus type [29]. Despite the extensive research on emotion recognition through facial expressions in ASC, most studies have either used still, rather than dynamic, stimuli [29, 30], allowed participants unlimited exposure to stimuli [31–33], or else limited their research to a narrow set of basic emotions [34, 35].

Another important channel of emotional information is prosody, which includes vocal intonation, stress, and rhythm [36]. Prosody plays an important role in linguistic functions as well as in emotional expression and comprehension [37, 38]. Whereas considerable research has documented the deficits children with ASC have in language production and comprehension, relatively few studies have examined the abilities of these children to recognize emotions through prosody [38, 39]. Studies on ER from prosody in individuals with ASC have yielded mixed results. Some studies have reported that adults or children with ASC experienced difficulties in using emotional prosody to identify the emotions of others [22, 36, 38, 40–43]. In contrast, several studies have reported that the emotional prosody perception in individuals with ASC is similar to that of TD controls [37, 44–47]. These mixed results could be due to methodological reasons [45], such as the use of cross-modal (e.g., face-voice) paradigms [48, 49] or the use of speech stimuli in which ER could rely on linguistic, rather than prosodic cues [3, 50].

A third channel of relevant emotional cues is body language, which includes gestures and postural changes. TD infants as young as 4 to 6 months show distinct attention to human biological motion [51]. At 2 years of age, children already rely on body language cues to predict the behavior of people they are watching [52], understand the goals of actions they observe [53], and anticipate people’s intentions [54]. At 3 years of age, children can verbally interpret biological motion, an ability that peaks by about 5 years of age [55]. Finally, children as young as 4 years successfully use body language to recognize social interactions, an ability that peaks by the age of 7 to 8 years [56].

In ASC, studies demonstrate difficulties discriminating biological from non-biological motion [57–59] and no preference for biological motion over object motion [60, 61]. Studies of emotional body language comprehension in ASC are scarce. The existing ones reported difficulties recognizing emotional, but not non-emotional motion from point-light displays in individuals with ASC, compared to TD controls [62, 63]. Children with ASC can distinguish social from non-social scenes, solely based on whole-body movements presented in point-light and stick-figure displays [56], but fail to interpret the nature of the actions presented, especially in the case of social scenes [56, 64–66]. Hence, the body motion recognition deficit in ASC appears to be restricted to percepts requiring mentalization and emotional interpretation [67–69].

Most studies that examined the ability of people with ASC to understand body language have either used stimuli such as point-light displays [67–69] or stick figures [56] or limited their investigation to a narrow set of basic emotions [24, 70]. It is therefore important to investigate whether this documented deficit exists in full body video stimuli, demonstrating a wider range of emotions, including complex ones.

In addition to the unimodal emotion-processing deficits described above, individuals with ASC show problems integrating information across multiple modalities [71]. Many of the atypical perceptual experiences reported by people with ASC may stem from an inability to efficiently filter, process, and integrate information from different modalities, when presented simultaneously [72].

Most studies that examine multimodal processing focus on the integration of auditory and visual social stimuli linked to communication, such as speech and its corresponding lip movements [73, 74]. The results of most of these studies indicate that the ability to integrate audiovisual social stimuli is impaired in individuals with ASC [75–78]. On the other hand, there is little research on the integration of information from facial expressions and body language or from multiple modalities in context. Multimodal integration ER studies reveal that presenting emotional cues in different channels does not necessarily help, and may even hamper the ability of individuals with ASC to recognize emotions and mental states [79, 80]. Previous studies of multimodal ER in ASC have shown deficits in adults [81] and in children [82]. However, the stimuli presented in these studies included verbal content, in addition to the visual and auditory emotional cues. Since individuals with ASC rely on verbal content as a compensatory mechanism [25], an examination of multimodal ER in children with ASC, in which stimuli have no verbal content to rely on, is desirable. In the absence of a linguistic component, such stimuli could also be used for cross-cultural comparison of ER in ASC.

As described above, studies showing no ER differences between TD individuals and individuals with ASC focus mostly, if not solely, on basic emotions [24, 34, 35, 70]. The six emotions referred to as “basic” (happiness, sadness, fear, anger, surprise, and disgust) are suggested to be cross-culturally expressed and recognized [83] and are to some extent neurologically distinct [84]. Unlike basic emotions, complex emotions are considered more context and culture dependent [85, 86]. They involve attributing a cognitive state as well as an emotion and may be belief- rather than situation-based [87]. The examination of basic ER in children with ASC yielded mixed results, with some studies reporting basic ER deficit in this group [21, 49, 88], whereas others reporting no difficulties in recognition of the basic emotions in children with ASC [25, 26, 28]. In contrast, studies investigating recognition of complex emotions and other mental states by children with ASC, compared to TD children, have shown more conclusive deficits [3, 89]. However, no study so far has conducted a direct comparison of basic and complex ER in children with ASC and their TD peers.

In terms of cross-cultural ER differences, meta-analyses have documented evidence for an in-group advantage, in that emotional expressions are more accurately recognized by individuals within the same culture, versus other cultures [90, 91]. However, individuals with ASC have been found to have poorer understanding of emotional cues (as shown above), as well as lower sensitivity to social cues [10] and lower social conformity [92]. Since individuals with ASC are less socio-emotionally sensitive within their own cultures, it is possible that cross-cultural differences in ER, if found, would be smaller in the ASC groups than in the TD groups.

The current study aimed to compare the recognition of the six basic and 12 complex emotions by children with ASC and TD controls. We examined ER unimodally through faces, voices, and body language, as well as multimodally through integrative scenarios with no verbal content and tested basic and complex ER cross-culturally in three different countries: Israel, Britain, and Sweden. We assessed differences between 5 to 9-year-old children with autism in the average IQ range and TD controls, comparable for age, sex, and IQ. We predicted that (a) the ASC group would have lower scores on the different ER tasks, compared to controls; (b) basic emotions would be recognized more easily than complex emotions; (c) the group differences would be greater for complex ER than for basic ER; (d) cultural differences between the three sites would be greater for complex ER than for basic ER; and (e) cultural differences on ER would be greater in the TD groups than in the ASC groups.

## Methods

### Instruments

#### Intelligence

Two subtests from the Wechsler Intelligence Scales, *Vocabulary* and *Block Design*, were used, representing verbal and performance IQ. In Britain and Sweden, subtests were taken from the locally standardized versions of the second edition of the Wechsler Abbreviated Scales of Intelligence (WASI-2) [93]. In Israel, they were taken from the fourth edition of the Wechsler Intelligence Scale for Children [94] and the third edition of the Wechsler Primary and Preschool Scale of Intelligence (WPPSI-3) [95], used according to the child’s age.

#### Autistic traits

The school-age form (4 to 18 years) of the Social Responsiveness Scale, second edition (SRS-2) [96], was used to assess the severity of autism traits. The SRS-2 measures social awareness, social communication, social motivation, social cognition, and inflexible behaviors applying a dimensional concept of autism. The SRS-2 includes 65 items, each scored on a four-point Likert scale, from 0 (“not true”) to 3 (“almost always true”), yielding a maximum of 195. It has demonstrated with good to excellent reliability and validity [96] and with good intercultural validity [97].

#### Facial affect recognition

*The Frankfurt Test and Training of Facial Affect Recognition* (FEFA-2) [98, 99] is a computerized test, examining facial ER of the six basic emotions (and neutral). The normed FEFA-2 test module comprises a series of 50 items for faces, showing good to excellent internal consistency and stability. The FEFA-2 was used in this study as a convergent validator for the test battery.

#### Emotion recognition battery

Emotion recognition was tested using four tasks, comprising facial expression videos, decontextualized vocal utterances, body language videos, and integrative video clips, presenting all three modalities in context. The battery is based on stimuli from several sources: The face task comprised 5-s long video clips from *Mindreading* (www.jkp.com/mindreading) [100]. The voice and body tasks comprised 1- to 3-s long audio clips and 4- to 24-s long video clips from *The EU-Emotion Stimulus Set* [101, 102]. The integrative task used 3- to 19-s long sampled scenes from old television series, following Golan et al. [82].

The tasks assessed ER for the six basic emotions (*happy*, *sad*, *afraid*, *angry*, *disgusted*, *surprised*) and for 12 complex emotions (*interested*, *bored*, *excited*, *worried*, *disappointed*, *frustrated*, *proud*, *ashamed*, *kind*, *unfriendly*, *joking*, *hurt*). Clips representing each emotion in all modalities were sampled from the above sources. Audio clips used neutral utterances (e.g., “what’s all this?”), spoken in appropriate emotional prosody. They were recorded in the native language on the three sites [101, 102]. In the body language clips, faces were masked, in order to prevent reliance on facial cues. In the integrative clips, the soundtrack was muffled, so that only paralinguistic information was available. Stimuli for the tasks were selected from an extended cross cultural ER survey, in which each stimulus was validated by 60 adults (20 from each site). In these surveys, participants had to recognize each stimulus by choosing the correct emotional label out of six options [101, 102]. Stimuli were considered valid if recognized correctly by at least 50% of the judges (*p* < .00001, binomial test). For each of the selected stimuli, the six-label scale was then reduced to four emotion labels—the target emotion label and three foils, in order to make it more suitable for children. One of the foils was always in the opposite valence to the target emotion (e.g., *afraid* as a foil for a *proud* target), and the other two were in the same valence as the target emotion (e.g., *excited* and *interested* as foils for a *proud* target). Label order was counterbalanced across items.

Next, item analysis was conducted with 20 TD children in Israel, 20 in Britain, and 10 in Sweden, aged 5 to 9 (half boys and half girls). Items were approved separately for each site, if the target answer was selected by at list 50% of the children in Israel and in Britain (*p* < .01, binomial test) and at least 60% in Sweden (*p* < .02, binomial test), and if none of the foils was selected by more than 40% of the children.

For the face task, 77.5% of the items met these criteria in Israel (with an average recognition rate of *M* = 74.0%, s.d. = 12.1%), 86.5% met them in Britain (recognition rate: *M* = 76.0%, s.d. = 12.4%), and 82.1% met them in Sweden (recognition rate: *M* = 80.0%, s.d. = 12.7%). For the voice task, 78.8% of the items met the criteria in Israel (recognition rate: *M* = 77.3%, s.d. = 13.6%), 91.7% in Britain (recognition rate: *M* = 75.8%, s.d. = 14.6%), and 89.47% met them in Sweden (recognition rate: *M* = 80.3%, s.d. = 10.7%). For the body task, 75.0% of the items met the criteria in Israel (recognition rate: *M* = 76.7%, s.d. = 9.3%), 90.3% in Britain (recognition rate: *M* = 81.3%, s.d. = 12.6%), and 85.3% met them in Sweden (recognition rate: *M* = 86.6%, s.d. = 9.9%). For the integrative task, 88.6% of the items met the criteria in Israel (recognition rate: *M* = 77.5%, s.d. = 12.2%), 95.5% in Britain (recognition rate: *M* = 81.34%, s.d. = 14.0%), and 87.5% met them in Sweden (recognition rate: *M* = 83.1%, s.d. = 13.7%).

Items that did not meet the inclusion criteria on the first item analysis round had their stimulus clip replaced, and the item analysis process was repeated until criteria were met. This resulted in slightly different stimuli being used in the face, body, and integrative tasks on the different sites (four items were different on the face task, one on the body task and one on the integrative task).

**Fig. 1:**
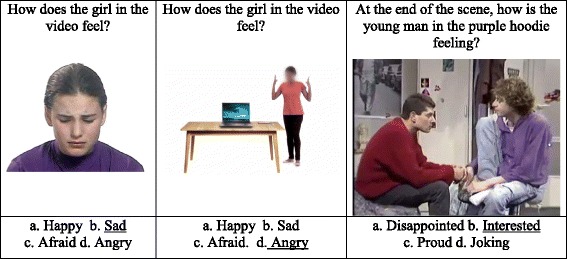
Screenshots (left to right) of the Face, Body, and Integrative tasks

Following these steps, four ER tasks were created (face, voice, body, and integration). In the face, voice, and integrative tasks, each emotion was represented by two clips, with a total of 36 items (score range 0–36) per task. The body gesture task included only 24 items (score range 0–24), two per emotion, comprising the six basic emotions and six of the complex emotions (proud, worried, excited, disappointed, frustrated, bored). The other complex emotions were not represented in this task, as they required more than one individual to convey the emotion (e.g., unfriendly). Figure 1 presents screenshots of the three visual tasks.

The ER battery was validated through correlation analysis between the four ER task scores and participants’ age, verbal and non-verbal abilities, level of autistic symptoms on the SRS-2, and FEFA-2 scores, as a convergent validator. Task intercorrelations were also computed. Bonferroni’s correction for multiple correlations was used. The correlations are presented in Table 1.

**Table 1:** Correlations of the emotion recognition tasks with background measures and with each other

	Face task	Voice task	Body task	Integrative task
Age	.48***	.51***	.44***	.47***
Vocabulary	.35***	.52***	.40***	.39***
Block design	.26**	0.12	0.14	0.17
SRS-2	−.41***	−.29**	−.38***	−.50***
FEFA-2	.54***	.49***	.46***	.49***
Voice task	.64***			
Body task	.61***	.68***		
Integrative task	.74***	.75***	.75***	

*p < .05, **p < .01, ***p < .001

As shown in the table, tasks were moderately intercorrelated (ranging between *r* = .61 and .75) and, in addition, had moderate positive correlations with participants’ age (ranging between *r* = .44 and .51) and verbal ability (ranging between *r* = .35 and .52). Non-verbal ability was only correlated with the face task scores (*r* = .26, *p* < .01). All tasks were negatively correlated with participants’ level of autistic symptoms (ranging between *r* = −.29 and −.50) and positively correlated with the FEFA-2 (range: *r* = .46–.54).

### Participants

The study was approved by the Psychology Research Ethics Committee at Cambridge University, by the Institutional Review Board at Bar-Ilan University, and by the Regional Board of Ethical Vetting Stockholm. Participants’ assent and parents’ informed consent were received before inclusion in the study. One hundred thirteen children, aged 5–9 years, took part in the study. The Israeli sample comprised 20 children in the ASC group and 22 in the TD group. The British sample comprised 16 children with ASC and 18 with TD. In Sweden, 19 children with ASC were compared to 18 children with TD. Participants with ASC had all been clinically diagnosed according to DSM-IV-TR or ICD-10 criteria [103, 104]. Diagnosis for children with ASC was corroborated by meeting ASC cutoff using the second edition of the *Autism Diagnostic Observation Schedule* (ADOS-2) [105]. Participants with ASC were recruited from volunteer databases, local clinics for children with ASC, special education classes and kindergartens, internet forums, and support organizations for individuals with ASC.

Participants in the TD groups were recruited from local primary schools and kindergartens. Parents reported their children had no psychiatric diagnoses or special educational needs, and none had a family member diagnosed with ASC. To screen out for ASC in the TD group, participants’ parents filled in the Childhood Autism Spectrum Test (CAST) [106]. None of the TD group participants scored above the cutoff of 15. Groups were comparable locally on age, gender, and standard scores of two subtests from the Wechsler Abbreviated Scale of Intelligence (WASI-2) [93]: Vocabulary and Block design. ASC groups and TD groups, separately, were comparable on SRS scores. The groups’ background data appears in Table 2.

**Table 2:** Participant demographics

	Israel			Britain			Sweden		
	ASC (*n* = 20)	TD (*n* = 22)	*t* (40)	ASC (*n* = 16)	TD (*n* = 18)	*t* (32)	ASC (*n* = 19)	TD (*n* = 18)	*t* (35)
Age	7.45 (1.31)	7.50 (1.47)	0.13	8.58 (1.03)	7.80 (1.42)	1.86	6.97 (0.67)	7.36 (1.2)	1.21
Vocabulary	11.15 (4.26)	11.82 (2.99)	0.59	11.38 (3.56)	12.22 (2.71)	0.79	9.05 (1.90)	10.11 (1.74)	1.76
Block design	12.5 (2.96)	11.55 (2.3)	1.17	11.44 (2.48)	9.72 (3.12)	1.76	11.00 (2.79)	11.83 (2.7)	0.92
SRS-2	74.46 (8.34)	42.93 (3.58)	12.59**	82.19 (7.57)	43.00 (5.42)	17.17 **	79.32 (12.06)	39.53 (1.42)	14.26**
			*χ* ^2^ (1)			*χ* ^2^ (1)			*χ* ^2^ (1)
Sex (M:F)	18:2	19:3	0.13	15:1	13:5	2.70	15:4	15:3	0.12

**p < .01

### Procedure

The research team met each child one to three times for assessment. In Israel, the meeting took place at the children’s homes. In Britain, some meetings were held at children’s homes and some at the Autism Research Centre in Cambridge. In Sweden, the meeting took place at the clinical research department KIND. All participants were tested individually. Parents filled out the SRS-2. The Wechsler subtests, the four ER computerized tasks, the FEFA-2, and ADOS-2 (children with ASC only) were administered to children.

The children were tested on the ER tasks in a counterbalanced form. Each task was preceded by two practice items. The experimenter read the instructions and the questions for all items, in order to avoid confounds due to reading difficulties. Optional answers were read out loud using neutral intonation, and the children were asked if they were familiar with all the possible answers. If the child was not familiar with a word, it was defined and demonstrated using a definition handout (e.g., *unfriendly: to be not nice to someone. John was unfriendly to Paul. He told him he didn’t want to play with him*). There was no time limit to answer each item, but participants could watch or listen to each clip only once. Completion of the whole ER battery took 1.5–2.5 h, including breaks.

### Statistical analysis

In order to examine differences between basic and complex ER in the different tasks, since the number of basic and complex emotions included in each task differed, accuracy proportion scores of basic and complex ER in the different tasks were computed for each participant. Average proportions of basic and complex ER task scores for the groups in the three countries appear in Table 3. After confirming the assumptions for MANOVA are met, a multivariate analysis of covariance (MANCOVA) with repeated measures was computed, with accuracy proportion scores of the four tasks (face, voice, body, and integrative) as the dependent variables, complexity (basic, complex) as the within-subject factor and group (ASC, TD), and country (Israel, Britain, Sweden) as the between-group factors. Since participants in the three countries differed on age and verbal ability, these two factors were entered as covariates. Pairwise comparisons with Bonferroni’s correction were used for further analysis of the interaction effects.

**Table 3:** Average proportions (s.d.) of basic and complex ER task scores for the ASC and TD groups in the three countries

	Israel				Britain				Sweden			
	Basic		Complex		Basic		Complex		Basic		Complex	
	ASC (*n* = 20)	TD (*n* = 22)	ASC (*n* = 20)	TD (*n* = 22)	ASC (*n* = 16)	TD (*n* = 18)	ASC (*n* = 16)	TD (*n* = 18)	ASC (*n* = 19)	TD (*n* = 18)	ASC (*n* = 19)	TD (*n* = 18)
Face task	.70 (.18)	.86 (.12)	.56 (.17)	.67 (.11)	.69 (.14)	.84 (.13)	.59 (.12)	.69 (.13)	.74 (.13)	.74 (.12)	.50 (.15)	.65 (.13)
Voice task	.68 (.22)	.73 (.13)	.57 (.24)	.72 (.15)	.64 (.14)	.74 (.16)	.68 (17)	.71 (.15)	.69 (.13)	.69 (.13)	.59 (.23)	.73 (.14)
Body task	.65 (.17)	.82 (.11)	.62 (.19)	.76 (.17)	.61 (.19)	.81 (.15)	.74 (20)	.80 (.18)	.69 (.17)	.76 (.16)	.57 (.23)	.67 (.19)
Integrative task	.72 (.20)	.85 (.14)	.59 (.20)	.77 (.11)	.71 (.20)	.84 (.11)	.61 (18)	.75 (.15)	.72 (.17)	.89 (.12)	.49 (.18)	.72 (.15)

## Results

### Overall analysis

The MANCOVA yielded an overall main effect for group (*F*[4,102] = 14.70, *p* < .001, *η* ^2^ = .37) and for complexity (*F*[4,102] = 10.49, *p* < .001, *η* ^2^ = .29) but not for country (*F*[8,204] = 1.73, n.s.). Both age (*F*[4,102] = 11.82, *p* < .001, *η* ^2^ = .32) and verbal ability (*F*[4,102] = 8.11, *p* < .001, *η* ^2^ = .24) had significant overall effects as covariates. The three two-way interactions: group by country (*F*[8,204] = 1.41, n.s.), group by complexity (*F*[4,102] = 1.56, n.s.), and country by complexity (*F*[8,204] = 1.90, n.s.) were not significant, but a three-way interaction of group by country by complexity came out significant (*F*[8,204] = 2.29, *p* < .05, *η* ^2^ = .08). In addition, complexity had a significant interaction with age (*F*[4,102] = 5.93, *p* < .001, *η* ^2^ = .19).

### Analysis per ER task

Effects of the analysis per ER task are detailed in Table 4. The analysis revealed the group, and complexity main effects found in the overall analysis were also significant for each and every task, with the TD group performing better than the ASC group over and above complexity and country, and with basic emotions recognized significantly better than complex emotions, over and above group and country. Age had a significant effect on all ER tasks, and verbal ability had a significant effect on all tasks but the face task. Similarly, the age by complexity interaction was significant for all ER tasks, with the exception of the face task. The three-way interaction of group by country by complexity came out significant only for the face task, suggesting this is the source for the interaction effect found in the overall analysis.

**Table 4:** Main effects, interaction, and covariate effects, which were significant in the overall analysis, detailed by ER task

		Face task	Voice task	Body task	Integrative task
Group	*F *(1,105)	33.53***	9.11**	28.13***	47.38***
	*η* ^2^	.24	.80	.21	.31
Complexity	*F *(1,105)	8.50**	8.98**	8.58**	31.08***
	*η* ^2^	.08	.08	.08	.23
Group × country × complexity	*F *(2,105)	6.50**	2.31	1.36	.02
	*η* ^2^	.11	.04	.03	.00
Verbal ability	*F *(1,105)	2.86	32.87***	6.17**	4.96*
	*η* ^2^	.03	.24	.06	.05
Age	*F *(1,105)	32.40***	22.52***	24.04***	24.18***
	*η* ^2^	.24	.18	.19	.19
Age × complexity	*F *(1,105)	1.37	9.88**	6.50*	12.86***
	*η* ^2^	.01	.09	.06	.11

*p < .05, **p < .01, ***p < .001

**Fig. 2:**
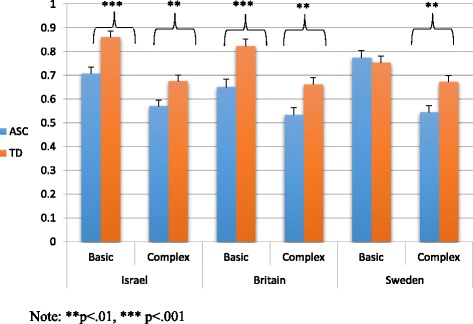
Basic and complex emotion recognition group differences on the face task in the three countries

Pairwise comparisons revealed the TD group performed significantly better than the ASC group in Israel and in Britain both for basic (Israel: mean difference = .15, s.e. = .04, *p* < .001; Britain: mean difference = .17, s.e. = .05, *p* < .001) and for complex (Israel: mean difference = .11, s.e. = .04, *p* < .01; Britain: mean difference = .13, s.e. = .04, *p* < .01) emotions. However, in Sweden, group differences were found for complex emotions (mean difference = .13, s.e. = .04, *p* < .01) but not for basic emotions (mean difference = .02, s.e. = .04, n.s.). Figure 2 illustrates these effects in the three countries.

An examination of the age by complexity interaction in the different ER tasks revealed the interaction was significant for all tasks, with the exception of the voice task. In order to further examine the interaction, bivariate correlation analysis, with Bonferroni’s correction, was conducted for age with basic and complex ER scores of each task, and the difference between the correlation of basic and complex emotions with age was compared for each task. The results, detailed in Table 5, show that, with the exception of the face task, age was more strongly correlated with complex ER than with basic ER for all tasks.

**Table 5:** Correlations of age with basic and with complex ER and the difference between them on the four tasks

	Basic	Complex	*t* (110)
Face task	.31**	.48**	2.12
Voice task	.25**	.50**	3.05**
Body task	.17	.51**	4.60***
Integrative task	.18	.52**	3.99***

**p < .01 ***p < .001

### Additional findings

In addition to the effects found in the overall analysis, some additional interaction effects were found for specific tasks. These will be described here, since there were specific hypotheses about them, which were not found over and above tasks. A group by complexity interaction was found only for the voice task (*F*[1,105] = 4.43, *p* < .05, *η* ^2^ = .04). Pairwise comparisons revealed participants with ASC (*M* = .61, s.e. = .02) scored lower than TD participants (*M* = .71, s.e. = .02) on complex emotion scores (mean difference = .10, s.e. = .03, *p* < .001) but not on basic emotion scores (ASC: *M* = .68, s.e. = .02; TD: *M* = .71, s.e. = .02; mean difference = .03, s.e. = .03, n.s.). In addition, a country by complexity interaction effect was found significant only for the body task (*F*[2,105] = 3.74, *p* < .05, *η* ^2^ = .07). Pairwise comparisons revealed that in Sweden, the body task scores for basic emotions (*M* = .75, s.e. = .03) were higher than for complex emotions (*M* = .64, s.e. = .03; mean difference = .11, s.e. = .04, *p* < .01). No difference between basic and complex body task scores was found significant in Israel (mean difference = .04, s.e. = .03, n.s.) and in Britain (mean difference = .04, s.e. = .04, n.s.).

## Discussion

The current study tested if there are differences in the recognition of basic and complex emotions between children with ASC and typically developing children across three countries: Israel, Britain, and Sweden. Emotion recognition (ER) was tested using dynamic facial expressions, body language, vocal expressions, and integrative scenarios. Children with ASC showed ER deficits in all three modalities and their integration in context. These deficits were found cross-culturally. Participants with ASC showed ER deficits in both basic and complex emotions. Cross-cultural agreement was found for all major findings, with minor deviations, reported below. Our findings highlight the multimodal nature of ER deficits in ASC, which exists for basic and complex emotions and is relatively stable cross-culturally.

As predicted, the ASC group had more difficulties in ER from facial expressions, body language, vocal expressions, and integrative scenarios, compared to the TD participants, even after controlling for age and verbal IQ. The significant group differences on task scores replicates previous findings of difficulties among individuals with ASC on emotion and mental state recognition from visual, auditory, and contextual stimuli [22, 24, 40, 43, 56, 66, 76–78]. These findings provide further support for ER and mentalizing deficits in children with ASC, which are evident cross-culturally.

As predicted, our findings show that basic emotions were recognized more easily than complex emotions on all modalities, regardless of child’s diagnosis. However, contrary to our hypothesis, group differences for complex ER were not greater than those found on basic ER. With the exception of an interaction effect between complexity and group in the voice task, our findings showed ER deficits in 5–9-year olds with ASC exist over and above complexity level. These findings match some reports on basic ER deficits in high-functioning children with ASC [23, 24, 35, 41–43, 70] and reports on complex ER deficits in this group [3, 40, 82]. The deficit we describe in the ASC group in basic ER, which was not found in some other studies (e.g., [28, 46]), may result from the age group tested. Whereas in older age, children with ASC may develop compensatory mechanisms allowing them to recognize basic emotions [25], our examination reveals a comprehensive ER deficit in 5–9-year-old children with ASC.

Another finding of developmental importance was the age by complexity interaction found in the current study. This interaction, found over and above group, revealed moderate correlations between age and ER in complex emotions, across all modalities, suggesting 5–9-year olds are still developing their ability to recognize complex emotions, comprising mental, as well as situational, aspects. Weaker correlations were found for age with basic ER in the voice, body, and integrative tasks, but not on the face task, suggesting basic ER skills have matured for at least some of the children in the tested age group. Interestingly, our findings show that the facial ER skills of basic and complex emotions alike continue to develop in this age group. In view of the centrality of facial expressions in ER, these findings provide an interesting example of the continuing development of facial expertise [9].

It is important to note that all of our visual stimuli used video clips, rather than still pictures. It is possible that processing of dynamic stimuli, which are more ecological in nature, may be more challenging to children with ASC, in comparison to still images. Specific deficits in processing of dynamic stimuli [107] may explain why our paradigm, which employed only dynamic stimuli in the visual channel, had demonstrated ER deficits in basic, as well as in complex, emotions in the visual tasks, but only complex ER deficits in the auditory task.

A striking finding in our study was the lack of major cultural differences. Our hypotheses that complex ER would show greater cultural variability than basic ER and that TD children would show greater cultural variability than children with ASC were not supported. These findings stand in contrast with previous arguments about greater cultural diversity in complex ER [85, 86]. It is possible that such cultural differences would be more salient in older age groups, when the acquisition of culture-specific nuances has been completed.

However, some cultural differences were found on the face and body language tasks. For facial ER, children with ASC in Sweden had performed more poorly than TD controls only on complex emotions and not on basic ER. This was in contrast to the British and Israeli ASC groups, which showed facial ER deficits both for basic and complex emotions. In addition, a significant interaction was found between country and complexity in the body language task. The analysis revealed that regardless of diagnosis, participants in Sweden had more difficulties recognizing complex, compared to basic, emotions from body language. This difference was not found in Israel and Britain. It should be noted that the body task stimuli were filmed in Britain, comprising actors of various ethnicities. Whereas the basic, more automatic and cross-cultural emotions were easily recognized in Sweden, as they were in Britain and in Israel, the more subtle, complex manifestations of emotion in body language may have been more challenging for children in Sweden. An examination of body language emotions, performed by Scandinavian (in comparison to non-Scandinavian) actors should be conducted in order to support (or refute) this potential findings of an in-group advantage [108].

A few limitations of the study should be noted: The current study examined cultural differences in ER as part of a cross-cultural examination of differences between individuals with and without ASC. Whereas the sample size is sufficiently large for the main question of the study, a larger sample of TD individuals within each country may be needed in order to appropriately examine ER cultural differences in the general population. The examination of ER cultural differences (and ASC-TD differences) in the various modalities should also be extended to additional, non-western cultures, such as African, or Eastern cultures [90], as these may reveal greater cross-cultural differences than the ones reported here.

In the current study, we were unable to examine ER gender differences, due to the relatively small number of females in the ASC groups. Gender differences in ER were found among adult participants with ASC in some studies [35, 40] but not in others [109] A replication of the current study with larger samples could examine the existence of gender differences in children with ASC as well.

Another limitation lies in some methodological variability across the sites and more specifically to the administration of assessments at home vs. the lab. These differences had resulted from participants’ difficulties to attend the lab in Israel and in Britain (due to distance and to parents’ needs). Hence, the clinical teams traveled to children’s homes. Despite the attempts to maintain a standardized testing environment (e.g., standardized protocol, individual assessment in a quiet room), it is possible that the different location for assessments had affected the results of this study. However, since the ASC and TD groups within each country were tested under similar conditions, it is unlikely that the different testing conditions have affected the group differences. Nonetheless, we recommend further studies to endorse more standardized structure if possible.

## Conclusions

Our findings demonstrate a supra-modal emotion recognition deficit in children with ASC, cross-culturally, for basic and complex emotion alike. Although the ER skills of children with ASC improve with age, like their TD peers, this overall ER deficit persists and calls for interventions [110, 111] which might narrow the developmental ER gap between children with ASC and their TD peers at the earliest possible stage.

## Abbreviations

ASC: Autism spectrum conditions
ER: Emotion recognition
TD: Typical development
